# Dual-chamber versus single chamber pacemakers, a systemic review and meta-analysis on sick sinus syndrome and atrioventricular block patients

**DOI:** 10.1016/j.heliyon.2023.e23877

**Published:** 2023-12-18

**Authors:** Abdul Rehman Shah Syed, Abdullah Akram, Muhammad Shaheryar Azam, Ayesha Irshad Ansari, Muhammad Ali Muzammil, Abdul Ahad Syed, Shaheer Ahmed, Syeda Javeria Zakir

**Affiliations:** Dow University of Health Science (Medicine), Pakistan

**Keywords:** Sick-sinus syndrome, Atrioventricular block, pacemaker, atrial fibrillation, Heart failure care

## Abstract

**Aims:**

The atrioventricular block (AVB) is a conduction system problem that results from the impairment in the transmission of an impulse from the atria to the ventricle, the disease has many etiologies. This article aimed to evaluate the efficacy and safety of dual and single-chamber pacemakers in patients with SSS and AVB.

**Methods:**

An electronic search of PubMed (Medline), EMBASE, and Google Scholar was performed from 2000 till August 15th, 2022. Retrieved articles were exported to Endnote Reference Library Software, where duplicate studies were removed from the list, and only articles meeting the eligibility criteria of this study were selected. RevMan 5.4 and STATA 16 software were used for the analysis. The modified Cochrane Collaboration's risk of bias and New-castle Ottawa scale were used for quality assessment of RCTs and observational studies respectively.

**Results:**

This study is composed of 8953 patients with sick-sinus syndrome and atrioventricular block. A total of thirteen outcomes are included in this meta-analysis, out of which atrial fibrillation significantly favored dual chamber [OR = 1.29; 95 % CI = 1.05–1.59; P = 0.01 I^2^ = 29 %] and overall complications [OR = 0.48; 95 % CI = 0.29–0.77; p = 0.03 I^2^ = 0 %] and pneumothorax [OR = 0.31; 95 % CI = 0.10–0.93; p = 0.04, I^2^ = 0 %] were satisfied by single-chamber pacing.

**Conclusion:**

This study concluded that neither single-chamber nor dual-chamber pacemakers are superior to each other, but they are unique in their own ways as the results of this study manifest remarkable reduction in atrial fibrillation rates and pneumothorax using dual-chamber and single-chamber pacemakers respectively.

## Highlights

• Dual-chamber pacing is more effective in reducing the adverse outcomes. However, no significant differences were found.
• This meta-analysis compared the efficacy and safety of dual and single-chamber pacemakers used in patients with heart block.
• Further trials comparing conventional pacemakers to leadless pacemakers are required in improving quality of life and safety.

## 1 Introduction

Sinus node dysfunction (SND) is an umbrella term for a variety of disorders in the sinus node and atrial impulse production and propagation. These include prolonged sinus bradycardia, sinus pauses, sinus arrest, and sinoatrial exit block as well as paroxysmal or permanent sinus arrest with substitution by subsidiary escape rhythms in the atrium, AV junction, or ventricular myocardium [1,2]. Bradycardias are accompanied by alternating patterns of tachycardia in around 50 % of instances, a condition known as tachycardia bradycardia syndrome [2]. One in every 600 cardiac patients over the age of 65 suffers from this disorder [3]. The incidence of Sick sinus syndrome (SSS) increases with age with almost equal proportions in both genders impacted, and is more prevalent in white people than black people [4]. SSS symptoms might include general symptoms like palpitations, fainting, lightheadedness, dizziness, fatigue, or cognitive impairment, with syncope being the most worrisome indication. Typically, a transient sinus arrest that results in a catastrophic reduction in cerebral blood flow is the primary cause of these symptoms [5], [6], Moderate gastrointestinal issues, intermittent oliguria or edema, and mild intermittent dyspnea are among some rare symptoms that could be associated with this condition. SSS may be related to symptoms brought on by the progression of conditions such as cerebral vascular accident, angina pectoris, congestive heart failure, dysrhythmia-induced emboli, peripheral thrombosis, and stroke [7]

The atrioventricular block is another conduction system problem that results from the impairment in the transmission of an impulse from the atria to the ventricle and there are many etiologies that could cause this disease. It is divided into three categories: First-degree AV block is defined as a simple sinus rhythm delay. Second-degree AV block (Wenckebach AV block), which results from depressed AV nodal conduction, is identified by a lengthening PR interval that ends in a dropped beat. Third-degree AV block, or complete AV block, is defined as the absence of all P waves [8].

A normal heartbeat consists of a synchronized contraction of the atrium and ventricle as well as a predictable sequence of ventricular activation using the specialized cardiac conduction system. In the absence of normal conduction, artificial pacemakers cause a variable-altered pattern of contraction [9]. Therefore, in patients with sick sinus syndrome, with chronic atrial fibrillation with atrioventricular block, sinus bradycardia with infrequent pauses or unexplained syncope with abnormal electrophysiological findings, or in normal sinus rhythm with second- or third-degree AV block, the symptoms can be adequately controlled with any pacemaker—a single chamber atrial, single chamber ventricular, or dual chamber pacemaker [10,11]. Permanent cardiac pacing will enhance clinical outcomes when there is clear evidence that a symptom is associated with SND. The association between symptoms and bradycardia is regarded as the gold standard of diagnosis and offers the best probability of therapeutic response [12].

As implied by the name "single-chamber pacemaker," pacing and sensing take place in only one chamber (atrium or ventricle). Pacing or sensing in both the atrium and ventricle is implied by a “dual-chamber pacemaker” [13]. In contrast to dual-chamber pacing, which restores atrioventricular synchronization and matches the ventricular pacing rate to the sinus rhythm, single-chamber ventricular pacing only avoids bradycardia and mortality from a ventricular standstill. As a result, as compared to single-chamber ventricular pacing, dual-chamber pacing enhances hemodynamic performance [14,15]. In 2009, more than 700,000 new pacemakers were reported to have been implanted worldwide [16]. Annually, roughly 105000 pacemakers are implanted in the US, with dual chamber pacemakers accounting for about 30 % and single chamber pacemakers for about 68 % of them [17]. Patients with chronic atrial fibrillation with AV block, who account for 15 %–30 % of all pacemaker recipients in Western countries, are the main benefactors of right ventricular single-chamber pacemakers. The most recent guidelines for single-chamber pacing, however, also advise considering elderly patients with complete AV block and low levels of activity, as well as patients with sinus node dysfunction and sporadic pauses [15].

The primary goals of pacemaker therapy are to extend a patient's life expectancy and improve their quality of life. In the current era of pacemaker technology, complications that might occur after pacemaker installation are common. About one in ten people who have cardiac pacemakers implanted experience difficulties [17]. Subcutaneous device pockets and transvenous connections are frequently the sources of complications. These include endocarditis, venous blockage, systemic infection, lead dislodgement, pneumothorax, and pocket hematoma [[18], [19], [20]]. This extensive systematic review and meta-analysis comprehensively assesses important outcomes encompassing various cardiovascular and procedural areas to evaluate if pacemakers dual-chamber or single-chamber benefits patients with SSS and AV. These outcomes include atrial fibrillation, congestive heart failure, stroke, death from all causes, including cardiovascular causes, hospitalization due to heart failure, and overall complications. We also investigate procedure problems such as pneumothorax and atrial lead dislodgement, providing information on their frequency and importance. Furthermore, this meta-analysis broadens its scope to include a quality-of-life evaluation to assess the overall impact of the therapies under discussion. By highlighting the interdependence of these outcomes and their consequences for clinical decision-making, this study attempts to assess the body of extant research thoroughly.

## 2 Methods

### 2.1 Data sources and search strategy

**Fig. 1:**
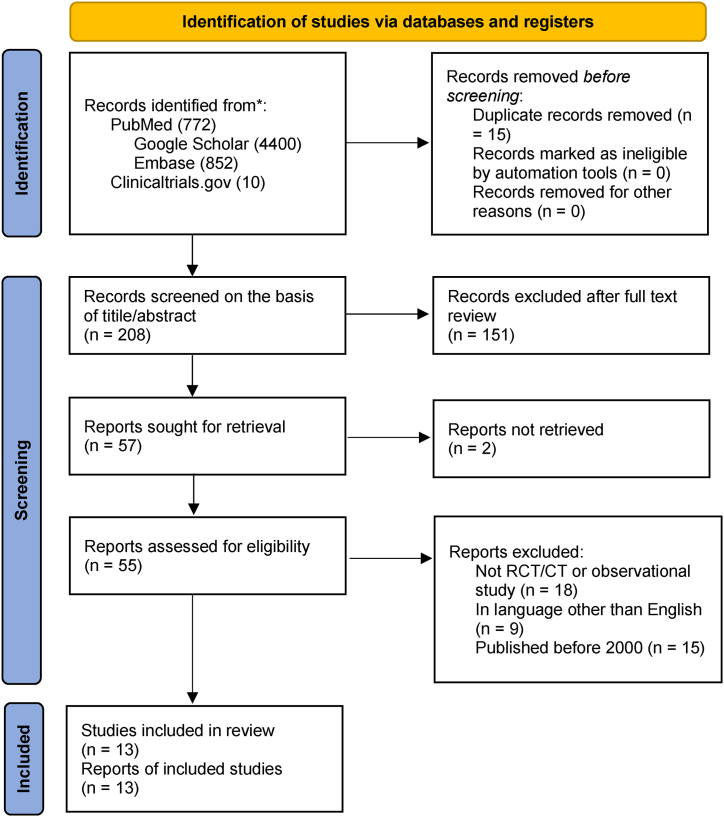
Prisma flow chart.

This meta-analysis was conducted in concordance with the Preferred Items for Systematic Reviews and Meta-Analysis (PRISMA) guidelines [21]. (Fig. 1)

**Fig. 2:**
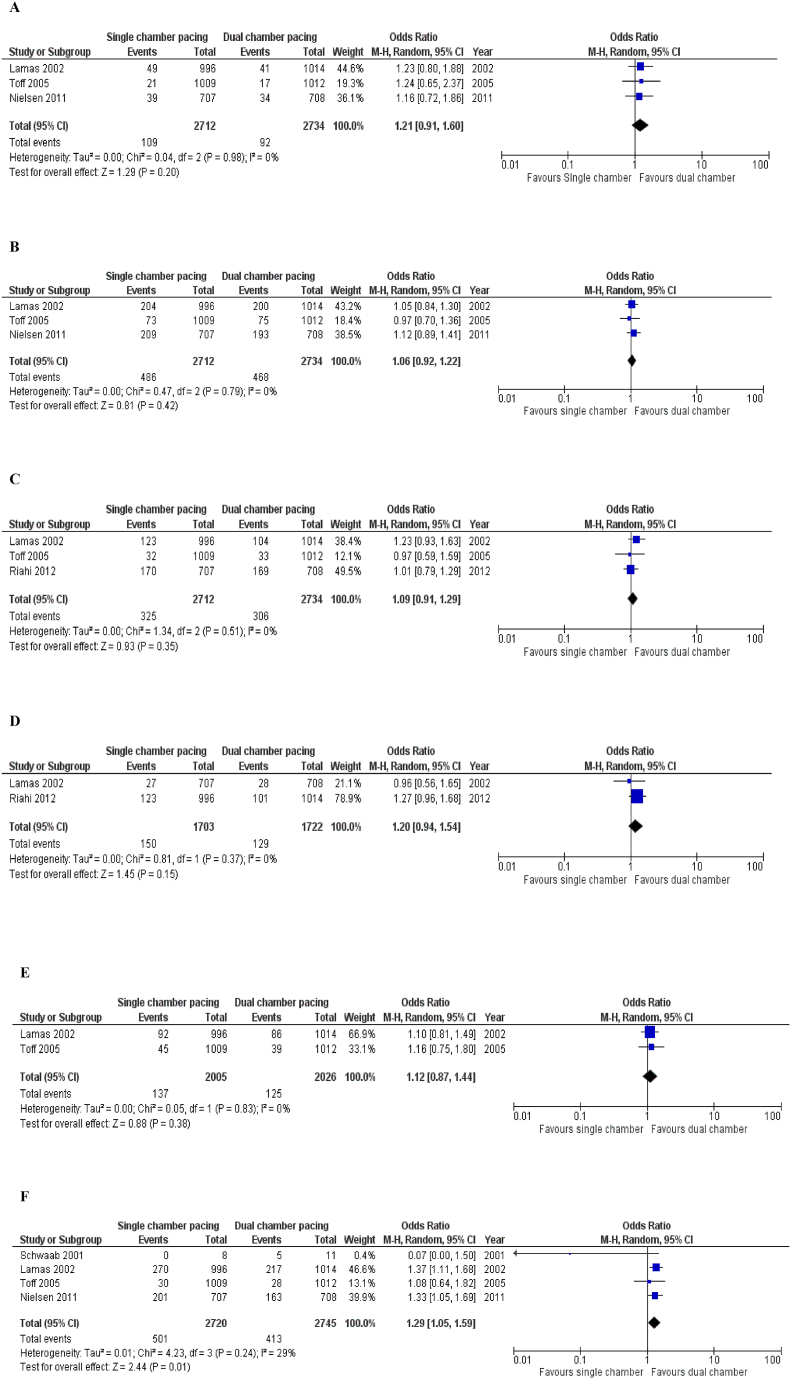
Forest Plots of Outcome of Included Clinical Trials 2 A = Stroke, 2B= All-cause mortality, 2C= Heart failure, 2D = Hospitalization due to heart failure, 2E = Mortality due to cardiovascular disease, 2F= Atrial fibrillation.

An electronic search of PubMed (Medline), EMBASE, and Google Scholar was performed from 2000 till August 15th, 2022, by two independent reviewers (SARS and AA), without any language restrictions. Furthermore, we also searched “clinicaltrials.gov” for any relevant published or unpublished clinical trials. In addition to this, we manually screened the reference list of included studies, similar meta-analyses, and review articles to include potentially relevant studies. Medical Subject Headings (MESH terms) were used to formulate the search strategy (Supplementary Table 1).

### 2.2 Study Selection

The eligibility criteria were; (1) Randomized controlled trials (RCTs) and observational studies like retrospective and prospective cohort and case-control studies with a target population comprising of elderly patients suffering from either sick sinus syndrome or atrioventricular block. (2) Single-chamber pacemaker being compared with a dual-chamber pacemaker, (3) at least 1 cardiovascular outcome reported.

While the case reports, review articles, expert opinions, comments, cross-sectionals, editorials, and studies published before the year 2000, were excluded from the analysis.

### 2.3 Data Extraction and assessment of study quality

Retrieved articles were exported to Endnote Reference Library Software, where duplicate studies were removed from the list. The remaining articles were then thoroughly assessed by the two independent reviewers (SARS and AA) and only those articles meeting the aforementioned eligibility criteria were included. All articles were first shortlisted based on title and abstract. A third reviewer (MSA) was consulted to resolve any disparity in the result. Data were extracted for the baseline characteristics and outcomes on an online Microsoft excel sheet from the finalized RCTs and observational studies. Baseline characteristics included were; year of publication; study design; sample size; the mean age of participants in both groups; pacing modes of dual and single chamber pacemakers; and the percentage of participants presenting with Coronary artery disease (CAD), Diabetes Mellitus (DM), and hypertension (HTN) at baseline. Following outcomes were included in this meta-analysis; atrial fibrillation; all-cause mortality; mortality due to CVDs; heart failure; hospitalization for heart failure; stroke; pacemaker syndrome; overall complications; pneumothorax; atrial lead dislodgement and quality of life.

The modified Cochrane Collaboration's risk of bias [22] tool was used by the two independent reviewers (SARS and MAM) to assess the quality of included trials shown in Supplementary Fig. 1. They also assessed the quality of observational studies using the Newcastle-Ottawa scale (Supplementary Table 4).

### 2.4 Statistical analysis

RevMan (Version 5.4. Copenhagen: The Nordic Cochrane Centre, The Cochrane Collaboration, 2014) and STATA software (version 16.0; STATA Corporation, College Station) were used for the statistical analysis. Forest plots were computed for the visual display of results. Using the random effects model, the results were reported as odds ratios (OR) with a 95 % confidence interval. Heterogeneity among studies was calculated by Higgin I^2^ and a value of greater than 75 % was considered high for I^2^, if the I^2^ value reported was greater than 75 %, the outcome was subjected to sensitivity analysis or meta-regression to determine the individual effects of each study on a certain pooled outcome.

### 2.5 Publication bias

**Fig. 3:**
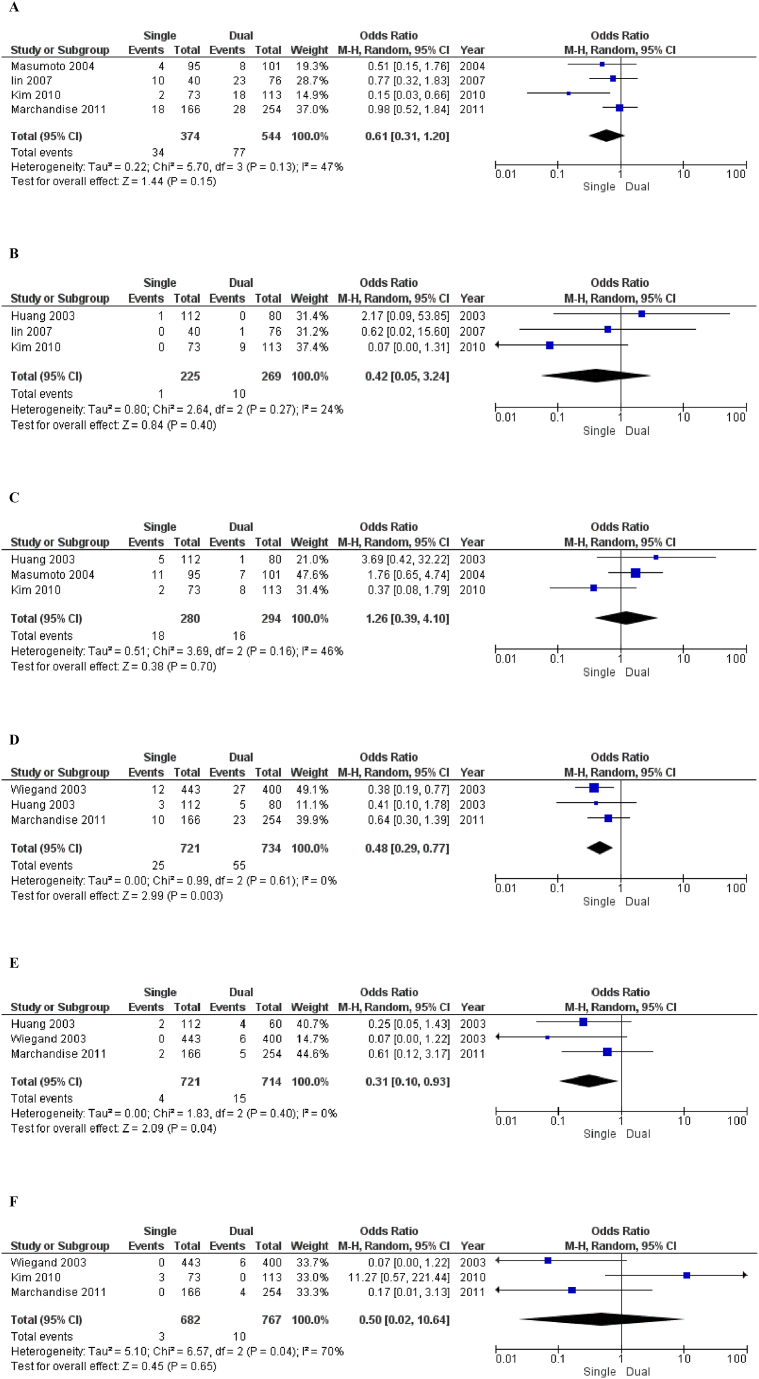
Forest Plots of Outcomes of Included Observational Studies 3 A = Atrial fibrillation, 3B= Congestive heart failure, 3C= All-cause mortality, 3D = Overall complication, 3E = Pneumothorax, 3F= Atrial lead dislodgement.

Funnel plots were created for the visual assessment of publication bias and are shown in Supplementary Fig. 2 for included RCTs and Fig. 3 for included observational studies. Egger's test was performed using comprehensive meta-analysis software. All outcomes were subjected to Egger's test except “mortality due to CVDs” and “hospitalization for heart failure” as these outcomes were not reported by 3 or more studies so they were not eligible for Egger's test. A p-value of <0.05 was considered significant (Supplementary Table 5 and Table 6 for RCT and observational studies respectively).

## 3 Results

### 3.1 Literature search results

A comprehensive search of three databases i.e. PubMed (Medline), EMBASE, and Google Scholar produced 208 results. After looking at the titles and abstracts, the study's eligibility criteria were taken into consideration. The remaining records were read through in their entirety. In the end, seven RCTs and six observational studies were chosen for meta-analysis and systematic review (Fig. 1).

### 3.2 Demographics

A total of 7000 SSS/AV block patients were randomized in the 7 trials, with 3487 in the single chamber pacemaker arm and 3513 in the double chamber pacemaker arm. In addition, six observational studies were selected for statistical analysis consisting of 1953 patients (929 with single chamber pacing and 1024 with dual chamber pacing). The mean ages of patients ranged between 57 and 77 years. The baseline characteristics of all included trials and observational studies were summarized in Table 1 and Table 2 respectively. The details like inclusion/exclusion criteria, primary outcome, follow-ups, and treatments used were also reported in Supplementary Table 2 for included trials and Supplementary Table 3 for observational studies.

**Table 1:** Baseline characteristics of included clinical Trails.

Study	No. of patients			Pacing modes	Age (Mean ± SD)			Female (%)			DM (%)			Stroke (%)			CAD (%)			HTN (%)		
	Single	Dual	Total		Single	Dual	Total	Single	Dual	Total	Single	Dual	Total	Single	Dual	Total	Single	Dual	Total	Single	Dual	Total
Schwaab 2001 [23]	8	11	19	AAIR vs DDDR	N/A	N/A	70 ± 7	N/A	N/A	38.09	N/A	N/A	N/A	N/A	N/A	N/A	N/A	N/A	N/A	N/A	N/A	N/A
Lamas 2002 [24]	996	1014	2010	ventricular pacing vs dual chamber pacing	74 ±8.8	74 ±9.6	N/A	48	47	N/A	20	24	N/A	11	11	N/A	24	28	N/A	61	63	N/A
Toff WD 2005 [15]	11009	11012	2021	–	79.9 ±6.0	79.9± 6.1	N/A	43.3	42.8	N/A	10.7	13.5	N/A	6.1	5.4	N/A	15.5	12.8	N/A	31.5	35.7	N/A
Ouali S 2009 [25]	30	30	60	VVIR vs DDD	N/A	N/A	76.5 ± 4.3	N/A	N/A	73.3	N/A	N/A	6.7	N/A	N/A	6.7	N/A	N/A	6.7	N/A	N/A	63.3
Nielsen JC 2011 [26]	707	708	1415	AAIR vs DDDR	73.5 ±11.2	72.4 ±11.4	N/A	66.8	62.3	N/A	9.6	10.2	N/A	8.6	7.5	N/A	13.3	12.7	N/A	34.1	33.8	N/A
Riahi S 2012 [27]	707	708	1415	AAIR vs DDDR	73.5 ±11.2	72.4 ±11.4	N/A	67	62	N/A	N/A	N/A	N/A	N/A	N/A	N/A	13	13	N/A	34.1	34	N/A
Kılıçaslan B 2012 [28]	30	30	60	VVIR vs DDD	N/A	N/A	68.9 ±6.9	N/A	N/A	46.6	N/A	N/A	30	N/A	N/A	N/A	N/A	N/A	20	N/A	N/A	60

**Table 2:** Baseline characteristics of included observational studies.

Study	No. of patients			Age (Mean ± SD)		Female (%)		DM (%)		CAD (%)		HTN (%)	
	Single	dual	Total	Single	Dual	Single	Dual	Single	Dual	Single	Dual	Single	Dual
Huang M 2003 [29]	112	80	192	70 ± 13	63 ± 16	40	30	N/A	N/A	N/A	N/A	N/A	N/A
Weigand UK 2003 [30]	443	400	843	76 ± 10	69 ± 13	46	41.5	N/A	N/A	65.5	58.5	N/A	N/A
Masumoto H 2004 [31]	95	101	196	63.1 ± 12.5	60.9 ± 14.1	68.4	54.4	N/A	N/A	57.1	55	N/A	N/A
Lin JM 2007 [32]	40	76	116	71 ± 10	67 ± 15	57.5	54.6	15	15	13	4	50	50
Kim WH 2010 [33]	73	113	186	58.9 ± 13.6	58.9 ± 13.6	61	49	11	10.6	5.5	8.8	31.5	30.1
Merchandise S 2011 [34]	166	254	420	77 ± 13	75 ± 15	41	32.3	27.1	21.7	23.5	15.7	55.4	55.1

### 3.3 Outcomes

A total of thirteen outcomes were analyzed in this meta-analysis.

#### 3.3.1 Outcomes of included RCTs (see Table 3)

**Table 3:** Outcomes of included clinical trials.

Study	Number of Patients			Atrial Fibrillation (%)		All-Cause Mortality (%)		Mortality due to CVDs (%)		Heart Failure (%)		Hospitalization for Heart Failure (%)		Stroke (%)	
	Single	Dual	Total	Single	Dual	Single	Dual	Single	Dual	Single	Dual	Single	Dual	Single	Dual
Schwaab 2001 [23]	8	11	19	0	45	N/A	N/A	N/A	N/A	N/A	N/A	N/A	N/A	N/A	N/A
Lamas 2002 [24]	996	1014	2010	27.1	21.4	20.5	19.7	9.2	8.5	12.3	10.3	12.3	10.3	4.9	4
Toff WD 2005 [15]	1009	1012	2021	3.05	2.8	7.2	7.4	4.5	3.9	3.2	3.3	N/A	N/A	2.1	1.7
Ouali S 2009 [25]	30	30	60	N/A	N/A	N/A	N/A	N/A	N/A	N/A	N/A	N/A	N/A	N/A	N/A
Nielsen JC 2011 [26]	707	708	1415	28.4	23	29.6	27.3	N/A	N/A	N/A	N/A	N/A	N/A	5.5	4.8
Riahi S 2012 [27]	707	708	1415	N/A	N/A	N/A	N/A	N/A	N/A	24	23.9	3.8	4	N/A	N/A
Kılıçaslan B 2012 [28]	30	30	60	N/A	N/A	N/A	N/A	N/A	N/A	N/A	N/A	N/A	N/A	N/A	N/A

##### 3.3.1.1 Stroke (Fig. 2A)

Stroke was reported in three of the seven [15,24,27] studies being analyzed (Single-chamber pacing: Patients 2712, Events 109, Dual-chamber pacing: Patients 2734, Events 92). The Forest plot showed that the dual chamber population is favored. Statistically, there was no significant difference in stroke [OR = 1.21; 95 % CI = 0.91–1.60; p-value = 0.20; I^2^ = 0 %] among single and dual chamber populations. No in-study heterogeneity was observed.

##### 3.3.1.2 All-cause mortality (Fig. 2B)

Three of seven selected studies [15,24,27] reported all-cause mortality (Single-chamber pacing: Patients 2712, Events 486, Dual-chamber pacing: Patients 2734, Events 468). As per the plot illustration of all combined studies, dual-chamber pacing is favored. Statistical analysis of the studies implies that no significant difference in all-cause mortality [OR = 1.06; 95 % CI = 0.92–1.22; p-value = 0.42; I^2^ = 0 %] between the 2 groups was found. No in-study heterogeneity was observed.

##### 3.3.1.3 Heart failure (Fig. 2C)

Three studies [15,24,28] provided data on heart failure (Single-chamber pacing: Patients 2712, Events 325, Dual-chamber pacing: Patients 2734, Events 306). Blobbogram favors dual chamber pacing. Quantitative analysis of the studies involved suggests no significant difference in heart failure [OR = 1.09; 95 % CI = 0.91–1.29; p-value = 0.35; I^2^ = 0 %] among observed groups. No in-study heterogeneity was observed.

##### 3.3.1.4 Hospitalization due to heart failure (Fig. 2D)

Two studies [24,28] determined the frequency of hospitalization for heart failure (Single-chamber pacing-1703 patients, 150 events; Dual-chamber pacing-1722 patients, 129 events). According to the plot depiction of all combined studies, dual chamber pacing was preferred. A quantitative analysis of the data of these two studies revealed no statistically significant difference in hospitalization due to heart failure between single-chamber and dual-chamber pacemakers. [OR = 1.20; 95 % CI = 0.94–1.54; p = 0.15 I^2^ = 0 %]. No in-study heterogeneity was reported.

##### 3.3.1.5 Mortality due to cardiovascular diseases (Fig. 2E)

Two studies [15,24] provided data on mortality due to cardiovascular diseases (Single-chamber pacing, 2005 patients, 137 events; Dual-chamber pacing, 2026 patients, 125 events). A dual-chamber pacemaker is favored based on the forest plot. The quantitative analysis of these two trials' findings demonstrated no significant difference in CVD mortality [OR = 1.12; 95 % CI = 0.87–1.44; P = 0.38 I^2^ = 0 %] between patients receiving single ventricular chamber pacemakers and dual chamber pacemakers. Furthermore, no in-study heterogeneity was observed.

##### 3.3.1.6 Atrial fibrillation (Fig. 2F)

Atrial fibrillation was reported in four out of seven studies [15,23,24,27] included in this meta-analysis (Single-chamber pacing, 2720 patients, 501 events; dual-chamber pacing, 2745 patients, 413 events). Dual-chamber pacemakers significantly reduced the incidence of atrial fibrillation as compared to single-chamber ventricular pacemakers as shown by the forest plot. [OR = 1.29; 95 % CI = 1.05–1.59; P = 0.01 I^2^ = 29 %] In-study heterogeneity was found to be low.

##### 3.3.1.7 Quality of life (QoL)

Quality of life was observed in four out of seven studies [23,24,26,29]. Health-related quality of life (HRQoL) was assessed by the SF-36 test in the study conducted by Kilicaslan et al. Eight different parameters were evaluated for the physical function subscale and physical role subscale. Between the two groups, no significant difference was observed in scores of seven parameters used in SF-36 except pain, which was significantly increased in DDD pacing (p = 0.04. Schwaab et al. used four different questionnaires to assess the quality of life on the basis of symptoms experienced. The first questionnaire, evaluating self-perceived health status depicts an insignificant difference between single vs dual chamber pacing. Karolinska's questionnaire slightly favored single-chamber pacing, even though it was still insignificant. Results of physical capacity assessment (done by using the Specific Activity Scale questionnaire) and pacemaker syndrome were unchanged in both groups. Ouali et al. measured QoL using the SF-36 questionnaire which showed the best values in the dual-chamber pacing, specifically, the domains assessing emotional components of QoL i.e., mental health (p = 0.004), vitality (p = 0.002) and general health (p = 0.043) were significantly improved among dual chamber pacing populations. Lamas et al. designed a QoL questionnaire with the use of SF-36 scales, the time-tradeoff utility scores, and the Specific Activity Scale class. QoL assessments were performed at 3 and 12 months after enrollment and yearly thereafter. After 3 months both groups showed substantial improvement in physical role assessed by the SF-36 questionnaire. Overall, dual-chamber pacing provided significant improvements in health-related quality of life, as compared to single-chamber pacing.

#### 3.3.2 Outcomes of included observational studies (see Table 4)

**Table 4:** Outcomes of observational studies.

Study	No. of patients			Atrial Fibrillation (%)		All-cause mortality (%)		CHF (%)		Overall complications (%)		Pneumothorax (%)		Atrial Lead dislodgement (%)	
	Single	Dual	Total	Single	Dual	Single	Dual	Single	Dual	Single	Dual	single	Dual	Single	Dual
Huang M 2003 [29]	112	80	192	N/A	N/A	4.5	1.25	1	0	3	6	1.78	6.66	N/A	N/A
Weigand UK 2003 [30]	443	400	843	N/A	N/A	N/A	N/A	N/A	N/A	2.71	6.75	0	1.50	0	1.50
Masumoto H 2004 [31]	95	101	196	4	8	11	7	N/A	N/A	N/A	N/A	N/A	N/A	N/A	N/A
Lin JM 2007 [32]	40	76	116	25	30	N/A	N/A	0	1.3	N/A	N/A	N/A	N/A	N/A	N/A
Kim WH 2010 [33]	73	113	186	2.8	15.2	2.8	7.1	0	8.8	N/A	N/A	N/A	N/A	3	0
Merchandise S 2011 [34]	166	254	420	11.2	11.4	N/A	N/A	N/A	N/A	6.	9.1	1.20	2	0	1.60

##### 3.3.2.1 Atrial fibrillation (Fig. 3A)

Out of the six observational studies [[32], [33], [34], [35]], atrial fibrillation was reported by four studies (Single-chamber patients: 374, Events: 34, Dual-chamber patients: 544, Events: 77). Statistical analysis of these studies indicates that the single-chamber population is favored even though it is not significant [OR = 0.61; 95 % CI = 0.31–1.20; p = 0.15; I^2^ = 47 %]. At last, in-study heterogeneity was found moderately high.

##### 3.3.2.2 congestive heart failure (Fig. 3B)

Three of the selected studies [30,33,34] identified Congestive Heart Failure (Single-chamber pacing-225 patients, 1 event; Dual-chamber pacing-269 patients, 10 events). The forest plot revealed that the single-chamber population is preferred. There was no statistically significant difference [OR = 0.42; 95 % CI = 0.05–3.24; p = 0.40; I^2^ = 24 %] in CHF between the single and the dual-chamber pacemaker groups. The findings indicated low heterogeneity.

##### 3.3.2.3 All-cause mortality (Fig. 3C)

Three out of six observational studies [30,32,34] gave data on all-cause mortality (Single-chamber pacing-280 patients, 18 events; Dual-chamber pacing-294 patients, 16 events). According to the blobbogram, dual-Chamber is preferred. Quantitative analysis of the results of these three studies illustrated no significant difference in all-cause mortality [OR = 1.26; 95 % CI = 0.39–4.10; p = 0.70; I^2^ = 46 %]. Furthermore, moderately high in-study heterogeneity was found.

##### 3.3.2.4 Overall complications (Fig. 3D)

Three studies out of six observational studies [30,31,35] shortlisted for this study, provided data on overall complications (Single-chamber patients: 721, Events: 25, Dual-chamber patients: 734, Events: 55). An analysis of the given information was done that clearly signifies, favoring population with single-chamber [OR = 0.48; 95 % CI = 0.29–0.77; p = 0.03 I^2^ = 0 %]. No in-study heterogeneity was found.

##### 3.3.2.5 Pneumothorax (Fig. 3E)

Three studies [30,31,35] discussed pneumothorax as their outcome (Single-chamber patients: 721, Events: 4, Dual-chamber patients: 734, Events: 15). After analyzing the given data, it was seen that single-chamber pacing was favored significantly [OR = 0.31; 95 % CI = 0.10–0.93; p = 0.04, I^2^ = 0 %]. No in-study heterogeneity was found.

##### 3.3.2.6 Atrial lead dislodgement (Fig. 3F)

Only three studies [31,34,35] looked into the effect of atrial lead dislodgement (Single-chamber pacing: 682 patients, 3 events; Dual-chamber pacing: 767 patients, 10 events). Single-chamber pacemakers is favored as per the plot depiction of all studies combined. A quantitative examination of the findings from these three studies found no statistically significant difference between using single-chamber and dual-chamber pacemakers for atrial lead dislodgement [OR = 0.50; 95 % CI = 0.02–10.64; p = 0.65; I^2^ = 70 %]. The heterogeneity within the study was found to be considerably high.

## 4 Discussion

This review and meta-analysis aimed to update the most recent evidence showing that dual-chamber pacemakers are more effective in reducing adverse health outcomes than single-chamber pacemakers in patients with sick sinus syndrome and atrioventricular block.

This study is composed of six observational studies and seven clinical trials totaling 8893 patients with SSS/AV block patients. By observing the results in RCTs, only atrial fibrillation had significantly reduced incidence in dual-chamber pacing when compared with single-chamber pacing, while the incidence of stroke, all-cause mortality, heart failure, hospitalization due to heart failure, and mortality due to cardiovascular diseases were also increased in single-chamber pacing, however, the result was not significant. In observational studies, there were increased incidences of pneumothorax and overall complications in dual-chamber pacing while the incidence of congestive heart failure, all-cause mortality, atrial lead dislodgment, and atrial fibrillation did not differ significantly in both groups.

The quality of life (QoL) was assessed by using a unique questionnaire in which different aspects were scrutinized. The emotional aspect of QoL such as mental health or vitality and general health, which was measured by Ouali et al., showed significantly improved results using dual chamber pacemakers [25]. In the study conducted by Kilicaslan et al., eight different parameters were evaluated for physical function and physical role subscale, but only one parameter i.e., the pain was observed to be improved using a dual chamber pacemaker leaving all other seven parameters non-significant [28]. When comparing dual-chamber atrial pacing to single-chamber atrial pacing, a meta-analysis of two trials revealed no statistically significant difference in functionality, general well-being, or multifaceted quality-of-life indicators, including cognitive capacity, which corresponded to our findings [36].

Pacemakers are usually used to stabilize the bradycardia symptoms in patients with an impaired conducting system or dysfunctional sinus node but they can lead to inappropriate contraction of the heart and eventually ventricular tachyarrhythmia [35]. Atrial fibrillation is an abnormal activity of the atria that appears as rapid, disorganized, inefficient, and irregular contractions on an electrocardiogram (ECG). The pathology can occasionally result in heart failure or stroke, which would necessitate interventions like pacemakers to regulate the heart rate and rhythm [37]. Two authors in their respective studies supported a rationale that interatrial septum pacing is almost similar to dual chamber pacing and helps reduce the frequency of atrial fibrillation episodes and control the ventricular rhythm [38,39]. Dretzke et al. [40] in their study reported that the rate of atrial fibrillation was remarkably reduced with dual-chamber pacing compared to single-chamber pacing, as also demonstrated in this study. Moreover, undiagnosed and persistent atrial fibrillation is commonly correlated to embolic events like stroke, the condition can be prevented with anticoagulation and restoration of sinus rhythm [41]. Wei‐Da Lu 42 conducted a study in Taiwanese patients and discovered that atrial high-rate episodes of greater than 2 min duration are significantly associated with neurologic events and these episodes were frequently seen in patients implanted with dual-chamber pacemakers, the result of this study also insignificantly tailored toward dual-chamber pacing for this outcome. While there was no statistically significant difference in stroke risk between the two pacing modalities, Steven J. Edwards' [36] analysis showed that dual-chamber atrial pacing had a lower incidence of paroxysmal atrial fibrillation than single-chamber atrial pacing. Both of these conclusions align with those of our study.

Anne B.Curtis et al. [43] in their trial proved dual-chamber pacing as an alternative to single-chamber pacing for heart failure on the basis of increased left ventricular end-diastolic volume index. In contrast, the results of this study are insignificant to exhibit any associated improvement with either pacing mode as Mohamed Abdelrahman et al. [44] also concluded in their study that neither dual-chamber pacing nor single-chamber pacing is good enough to treat heart failure or all-cause mortality but they have found HIS-bundle pacing as a better substitute. The debate about using temporary or permanent pacemakers still continues but the decision depends on the patient's hemodynamic stability, those with right ventricular failure complicated by heart block reported substantial improvement in their hemodynamics with dual chamber pacing, which eventually amplifies the survival rate and improved the outcome of all-cause mortality in these patients [45]. Furthermore, the MOST study [46] showed a decrease in hospitalization for heart failure after pacemaker implantation, in contrast to our analysis results. In addition, different studies have also proven that 92 % of the myocardial infarction population deteriorated by heart block live longer with pacemaker therapy, further, these studies recommend that prophylactic implantation of pacemakers should be considered in this subgroup to prevent the complete heart block. This suggests that mitigating the risk of mortality due to any cause is not associated with any specific type of pacemaker as the results of this study exhibit [[47], [48], [49], [50], [51]]. Despite this, a different study found that people with single-chamber pacemakers had a 90-day mortality rate of 5 %, while people with dual-chamber pacemakers had a mortality rate of 3 % [52]. This finding contrasts with our own, which found no statistically significant link between pacemaker type and mortality.

Considering pneumothorax and lead dislodgement as an acute lethal consequence of post-implantation of pacemakers regardless of the choice of the vein used [53], R. K. Aggarwal [19] in their study found no variation between the types of pacemakers. On the other hand, single-chamber pacing showed a remarkably low probability of pneumothorax when compared to dual-chamber pacing as evidenced in this study and also supported by the study conducted by Shurrab et al. [54] Lead dislodgement is another complication, commonly encountered with single-chamber pacemakers. The rate of dislodgement depends on several factors such as lead design, the weight of the lead, imaging used during implantation, the physician implanting the pacemaker, and the chamber of the heart (common with atrial pacing) [[55], [56], [57]].This study, on the other hand, found no significant association between the two types of pacemakers.

## 5 Limitation

The observational type of this study has limited us to accessing patients individually and obliges us to rely on published data. The variation in the follow-up period between each study could lead to bias and make it difficult to determine the far-reaching consequences of different pacemakers. The difference in mean age between RCTs and included observational studies could be debated that the younger patients may be healthier and may show the improved outcome. In addition, the data for some outcomes was not sufficient enough to include them in our analysis which could affect the efficiency of this study. Lastly, the data about comorbidities was not provided sufficiently in the involved studies which could manipulate the overall outcome.

## 6 Conclusion

Finally, this study concluded that no pacemaker is superior to another, but they are unique in their own ways as the results of this study manifest remarkable reduction in atrial fibrillation rates and pneumothorax using dual-chamber and single-chamber pacemakers respectively. More randomized control trials are needed to be done in order to determine the effectiveness of dual chamber and single chamber pacing on patients’ quality of life, the effect on mortality, and other applicable uses of pacemakers. The point to make sure of in future trials is that the follow-up should be long enough to rationalize the long-term effects of different pacing modes. Finally, the modern era has revolutionized leadless pacemakers, further trials are needed to focus on comparing conventional pacemakers to leadless pacemakers in improving quality of life and safety [11].

## Data availability statement

Data will be available on request to the corresponding author

## CRediT authorship contribution statement

**Abdul Rehman Shah Syed:** Conceptualization, Formal analysis, Methodology, Writing - original draft, Writing - review & editing. **Abdullah Akram:** Conceptualization, Formal analysis, Methodology, Writing - original draft. **Muhammad Shaheryar Azam:** Conceptualization, Formal analysis, Methodology, Writing - original draft. **Ayesha Irshad Ansari:** Conceptualization, Formal analysis, Methodology, Writing - original draft. **Muhammad Ali Muzammil:** Data curation, Formal analysis, Writing - original draft. **Abdul Ahad Syed:** Syed, Conceptualization, Formal analysis, Writing - original draft. **Shaheer Ahmed:** Conceptualization, Methodology, Writing - original draft. **Syeda Javeria Zakir:** Conceptualization, Methodology, Writing - original draft.

## Declaration of competing interest

The authors declare that they have no known competing financial interests or personal relationships that could have appeared to influence the work reported in this paper.
