# Supplementary material for: Dual-chamber versus single chamber pacemakers, a systemic review and meta-analysis on sick sinus syndrome and atrioventricular block patients
Source: Heliyon. 2023 Dec 18;10(1):e23877. doi: 10.1016/j.heliyon.2023.e23877 (PMC10792191; doi:10.1016/j.heliyon.2023.e23877)

Supplementary Table 1: Search Strategy

Databases	Search Strings
**PubMed**	(single chamber pacemaker OR single-chamber pacing OR single-chamber pacemaker OR single chamber cardiac pacing device OR VVI OR ventricular demand pacing OR VVIR OR AAIR OR dual vs single chamber pacemaker) AND (dual-chamber pacing OR dual-chamber pacemaker OR Dual-Chamber Pacemaker Programming OR DDD OR Dual-Chamber Pacemaker OR DDI OR DDIR OR VDD OR DDT OR dual chamber pacemaker OR dual chamber cardiac pacing device OR Dual Chamber Rate Adaptive Pacemaker OR DDDR) AND (block OR atrioventricular block OR bradycardia OR sick sinus block OR sick sinus syndrome OR sinus dysfnction)
**Google Scholar**	Single chamber pacemaker OR dual chamber pacemaker OR atrioventricular block OR sick sinus syndrome
**Embase**	Single chamber pacemaker OR dual chamber pacemaker OR atrioventricular block OR sick sinus syndrome

Supplementary Table 2: Study Characteristics of Included RCTs

Characteristics	Schwaab [23]	LAMAS [24]	Toff WD [15]	OUALI S [26]	Nielsen JC [27]	Riahi S [28]	Kilicaslan B [29]
**Trial name**	AAIR Versus DDDR Pacing in the Bradycardia Tachycardia Syndrome	VENTRICULAR PACING OR DUAL-CHAMBER PACING FOR SINUS-NODE DYSFUNCTION	Single-Chamber versus Dual-Chamber Pacing for High-Grade Atrioventricular Block	DDD versus VVIR Pacing in Patients, Ages 70 and Over, with Complete Heart Block	A comparison of single-lead atrial pacing with dual-chamber pacing in sick sinus syndrome	Heart failure in patients with sick sinus syndrome treated with single lead atrial or dual-chamber pacing: no association with pacing mode or right ventricular pacing site	Comparison of DDD versus VVIR pacing modes in elderly patients with atrioventricular block
**Patients no.**	19	2010	2021	30	1415	1415	30
**Year of publication**	2001	2002	2005	2009	2011	2012	2012
**Trial type**	A prospective, randomized, double-blind, crossover trial.	Randomized control trials.	Randomized, parallel-group trial	A double-blind randomized two-period crossover study.	Randomized control trial.	Randomized control trial.	A randomized, two-period crossover study.
**Inclusion criteria**	Patients must be on antiarrhythmic medication for preventing atrial fibrillation or atrial flutter, all must have received a dual chamber pacing system for either medically induced symptomatic bradycardia or spontaneous bradycardia. They all must have experienced at least two documented paroxysms of atrial tachyarrhythmia. In addition, patients had to comply with each of the following definitions of chronotropic incompetence: peak	Patients were eligible if they were at least 21 years old and must be going under implantation of a dual-chamber, sinus node dysfunction that was rate modulated by a pacing system, and were in a sinus rhythm. They also must be able to score 17 or higher on the mini-mental state examination, and be eligible for quality-of-life analyses.	Patients of 70 and above this age group who were scheduled to have implantations of their first pacemaker for high degree AV block.	Patients with age greater than 70, patients with permanent complete heart block with permanent ventricular capture and a sinus rhythm on electrogram, 6MWD test is carried by the patients	Patients with symptomatic bradycardia, patients with pauses greater than 2 sec in sinus arrest or documented Sino-atrial block or sinus bradycardia less than 40 b.p.m for greater than 1 min while awake, age 18- 70 years and PR interval less than 0.22 s or Less than 0.26 s of PR interval and QRS width less than 0.12 s.	Patients with symptomatic bradycardia, patients with pauses greater than 2 sec in sinus arrest or documented Sino-atrial block or sinus bradycardia less than 40 b.p.m for greater than 1 min while awake, age 18- 70 years and PR interval less than 0.22 s or Less than 0.26 s of PR interval and QRS width less than 0.12 s	Patients with sinus rhythm and complete heart block before implantation on ECG who were able to perform daily activity tests.

	exercise heart rate, 100 beats/min,13 peak exercise heart rate, (220 2 age) 3 0.75,7 and heart rate at half the maximum workload, 60 1 2 beats/min per mL O2/kg/min.						
**Exclusion criteria**	Patients with any significant valvular heart disease or if they had a complete bundle branch block, a bi-fascicular block, or a PQ interval greater than 240 ms during sinus rhythm at rest. 24-hour Holter ECG was used to exclude second or third-degree AV block	Any patient with serious concurrent illness at the site was excluded.	Patients with New York Heart Association (NYHA) class IV heart failure, advanced cognitive dysfunction, total immobility, and advanced cancer (life expectancy of less than one year), and chronic established atrial fibrillation	Patients with sick sinus syndrome, chronotropic incompetence, and permanent atrial fibrillation.	Patients with panned cardiac surgery or a life expectancy shorter than 1 year or atrioventricular block; bundle branch block; long-standing persistent atrial fibrillation (.12 months); atrial fibrillation with ventricular rate, 40 b.p.m. for ≥1 min or pauses .3 s; a positive test for carotid sinus hypersensitivity.	Atrioventricular block; bundle branch block; long-standing persistent atrial fibrillation (.12 months); atrial fibrillation with ventricular rate, 40 b.p.m. for ≥1 min or pauses .3 s; a positive test for carotid sinus hypersensitivity.	Any patient with left ventricular dysfunction, acute coronary syndrome, sick sinus syndrome, hypertrophic obstructive cardiomyopathy, left ventricular systolic dysfunction, sinus bradycardia with AV block, Mobitz type 1 AV block, supraventricular tachycardia, and transient AV block
**Treatment**	AAIR or DDR Pacing with optimized AV delay	The pacemaker was randomly assigned with either rate-modulated dual chamber pacing or rate-modulated ventricular pacing	Single chamber ventricular pacing system or a dual-chamber system was assigned<br>The assignment to fixed-rate or rate-adaptive pacing was randomly determined within the single-chamber group.	Dual chamber pacemakers were inserted for complete AV block benefit significantly from DDD pacing compared with VVIR pacing.	Patients were randomized to DDDR pacing and additional lead was implanted in the right ventricle. A bipolar lead was implanted in the right atrium. For implanting an AAIR pacemaker, a 1:1 atrioventricular conduction was required. An atrial pacing test was performed at 100	Patients were randomized to DDDR pacing and additional lead was implanted in the right ventricle. A bipolar lead was also implanted in the right atrium. Frontal fluoroscopy plane was used to note the ventricular lead's position as being either apical or non-apical.	A non-permanent transvenous pacemaker was placed. Then dual chamber pacemaker was implanted and the patient was randomized to either VVIR or DDD modes.

					bpm in all patients.		
**Primary outcome**	AAIR and DDDR pacing were found to be equally effective in patients with bradycardia tachycardia syndrome caused by medicated or paced reasons, but due to safety reasons, DDDR pacing with optimized AV delay should be the preferred option.	Significant improvement was found in dual chamber pacing compared to ventricular pacing. Dual chamber pacing also reduces the risk of atrial fibrillation, reduces the signs and symptoms of the heart and may also increase the QoL.	During the first three years after implantation of a pacemaker, there was no association found in pacing mode and the rate of death in elderly patients with high-grade AV block from all causes during the first five years or the incidence of cardiovascular events.	DDD pacing was associated with improved QoL and systolic ventricular function in elderly patients compared with VVI pacing.	DDDR pacing was used in routine as there was no difference found in the death rate from any cause between AAIR pacing and DDDR pacing programmed with a moderately prolonged atrioventricular interval.	DDDR pacing was found to be safe in patients with SS without increasing the risk of HF.	Improvement in QoL and exercise performance was found in patients with active elderly patients with complete heart block and who were implanted with DDD pacing or VVIR pacing.
**Follow up**	Four weeks after implantation, patients were randomized to either AAIR or DDDR mode. Total of 6 months with 3 months apart switching the pacing mode.	Patients were followed four times during the first year and twice yearly thereafter.	Follow-up was minimum for 3 years with scheduled visits at 1, 4, 10, 16, and 36 months in which the assessment of pacemaker function and outcome of events were recorded.	Follow-up took place after 3 months in which their pacemakers were reprogrammed to the alternative mode for further 3 months. At the final visit, all patients were returned to DDD mode.	Follow-up took place after 3 months and again every year after implantation for up to 10 years	Follow-up took place after 3 months and again every year after implantation for up to 10 years	Follow-up took place after one month , when standard protocol tests were submitted, and the same tests were performed after another month.

Supplementary Table 3: Study Characteristics of Include Observational Studies

Characteristics	Huang M [30]	Wiegand UK [31]	Masumoto H [32]	Lin JM [33]	Kim WH [34]	Marchandise S [35]
**Trial Name**	Optimal Pacing for Symptomatic AV Block: A Comparison of VDD and DDD Pacing	Long-Term Complication Rates in Ventricular, Single Lead VDD, and Dual Chamber Pacing	Long-term clinical performance of AAI pacing in patients with sick sinus syndrome: a comparison with dual-chamber pacing	Long-term clinical performance of AAI pacing in patients with sick sinus syndrome: a comparison with dual-chamber pacing	Long-Term Outcome of Single-Chamber Atrial Pacing Compared with Dual-Chamber Pacing in Patients with Sinus-Node Dysfunction and Intact Atrioventricular Node Conduction	Long-term follow-up of DDD and VDD pacing: a prospective non-randomized single-center comparison of patients with symptomatic atrioventricular block
**Patients No.**	192	1214	196	116	186	254
**Year of Publication**	2003	2003	2004	2007	2010	2011
**Trial Type**	Retrospective study.	Retrospective study.	Retrospective study.	Retrospective study.	Retrospective study.	Prospective study.
**Inclusion criteria**	Patients were included if they had at least a period of one-year follow-up, should be inserted a pacemaker the first time, should be symptomatic second or third-degree AV block, and sinus function should be normal as judged by outpatient as well as in-patient referral material.	Patients were included if they had received a VVI(R), VDD, or a DDD(R) system with those that showed symptoms or with a high degree of disturbance in the conduction of AV nodal conduction.	Patients were included if they had right bundle branch block, AV block of the first degree, or unifascicular block	Patients were included if they were implanted with either AAIR or DDDR pacemakers and were regularly followed up at the clinic. The patients in the DDDR group had to spend> 80% of their time in VP with atrial synchronization while in the AAIR group, the patients must have spent >80% of their time in atrial pacing.	Patients with normal AV conduction, aged equal to or more than 20 years with sinus node dysfunction.	AV node conduction disorders with patients receiving VDD or DDD pacemakers were included.
**Exclusion Criteria**	Any underlying atrial fibrillation at implants, syncope found in any patients, any evidence of sinus node disease, or follow-up was less than one year	High-degree AV block that was symptomatic with permanent atrial fibrillation, Paroxysmal atrial fibrillation without evidence of sinus node dysfunction, and documented sinus node dysfunction in any patient, all were excluded from the study.	Any patients having spontaneous AV block of the second or third degree, complete left bundle branch block, or bifascicular block on their ECG were excluded	Depressed LV systolic function (EF< 50%), moderate or severe valvular heart diseases, significant coronary artery disease (>2 vessels, angiography proved), previous myocardial infarction, dilated cardiomyopathy or hypertrophic cardiomyopathy, chronic renal failure and chronic obstructive pulmonary disease, all were criteria for exclusion.	Patients with an atrial fibrillation suppression pacing algorithm along with a pacemaker were excluded. Also, any patients that underwent cardiac surgery or AV node ablation, had bundle branch block, multisite atrial or ventricular pacing, or AF.	Patients were excluded under the age of 18 years if they had a congenital heart disease if the ejection fraction was less than 35 %, any history of atrial fibrillation, or sick sinus node dysfunction at the time of implantation. Chronotropic insufficiency was another reason for exclusion if they were able to perform an exercise stress test

**Treatment**	VVD leads and DDD leads in which VVD leads were steroid-eluting passive fixation leads with 2 atrial sensing rings 11-15 cm from the tip of the lead while in DDD leads, it was fixed leads in both the ventricle and atrium that were eluted by steroids.	VVD pacemaker, DDD pacemaker as well as VVI systems, all were implanted in which DDD pacemaker and VVD pacemaker both were implanted in the ventricles with the DDD group receiving unipolar leads, while the VVI group, had an active-fixation mechanism with unipolar leads.	AAI pacemakers as well as DDD pacemakers were implanted in patients.	Single-chamber ventricular rate-modulated pacing (AAIR) pacemakers as well as Dual chamber rate-modulated pacing (DDDR) pacemakers.	Patients were implanted with AAI(R) pacemakers as well as DDD(R) pacemakers with normal AV conduction.	VVD and DDD pacemakers were implanted in which the VVD pacemaker was placed between the ventricular tip and the atrial sensing bipole while the DDD pacemaker was positioned under fluoroscopic control to achieve sensing values.
**Primary outcome**	Patients with AV-block of high degree and normal sinus node dysfunction with DDD pacemaker have an alternative to VVD pacing as it is highly reliable with lower cost and abbreviated implantation time.	Patients with AV block can be recommended with single lead VDD pacing as operation time and rates of complication are reduced in comparison to VVI pacing.	In SSS and normal AV conduction, AAI pacing which is a simple system with a single-lead and a single-chamber pacemaker can achieve an outcome similar to the DDD mode in a pacemaker.	Common pacemaker patients with normal baseline left ventricular function after chronic DDDR pacing may have abnormal heightening of plasma NT- proBNP concentration imposed by right ventricular apical pacing in Interventricular mechanical dyssynchrony.	In patients with SND and intact AV conduction, the preferred pacing mode was found to be AAI(R) pacing in comparison with DDD(R) pacing.	While comparing the VDD and DDD pacing groups, poor atrial signal detection without clinical impact was found in a large number of patients of VDD- paced patients. However, atrial under-sensing was not influenced by the incidence of atrial fibrillation, myocardial infarction, dilated cardiomyopathy, or mortality.
**Follow-up**	Follow-up was performed within a week of the implant. The hospitalized patients were followed under 1-3 days after implantation, while out-patients had a follow-up after 5-7 days of the implant. The next follow-up was done after 3 months, then 1 year, and then annually.	The follow-up period was around 63± 31 months with a rate of 12.4% death in patients.	The follow-up period was every 3-6 months. The average follow-up period was 8.7 (±6.2) and the range was 0.5-21 years in the AAI group while in the DDD group, it was an average of 7.6 (±4.7) and a range of 0.8-18 years.	N/A	The follow-up period was assigned to 1 month, 3 months, and then once every 6 months. If the patients missed a visit, the data were compiled through contact with the patient's family by nurses or clinical records.	The follow-up period was up to 24 hrs. 2 months post-implantation and then every 6 months by a rythmologist. Between the period of 1 June 2009 and 31 December 2009, all patients were informed to come and undergo a final control visit which included a full medical examination, 12 ECG lead monitoring, and many more.

Figure 1: Quality assessment using the Revised Cochrane Risk of Bias (RoB) tool

Figure 1A:

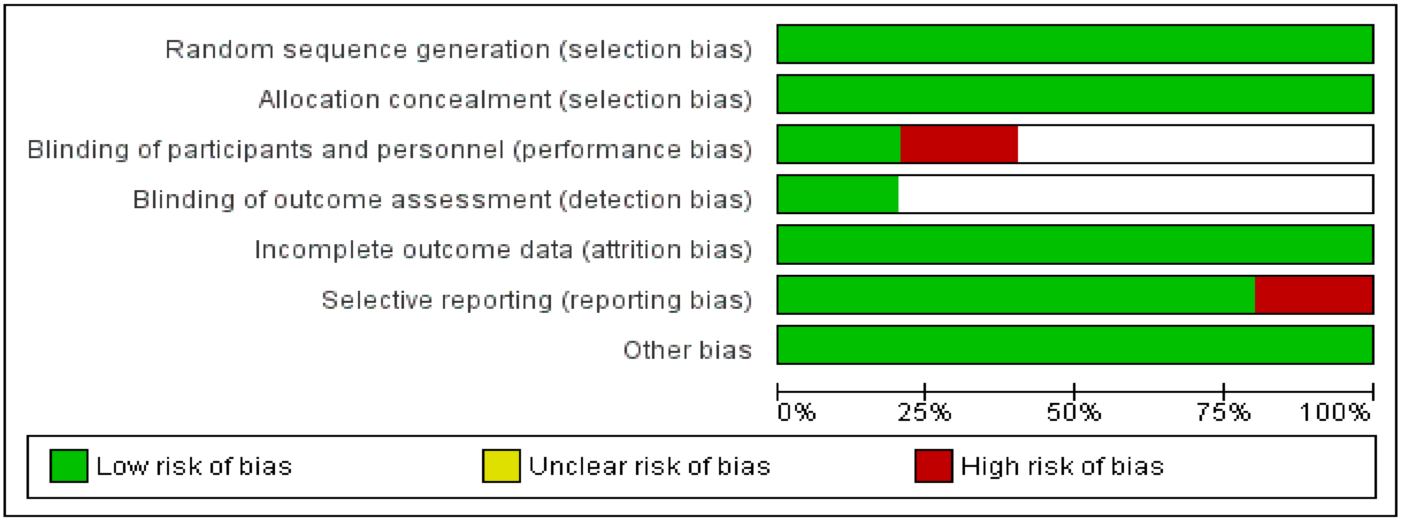

Figure 1B:

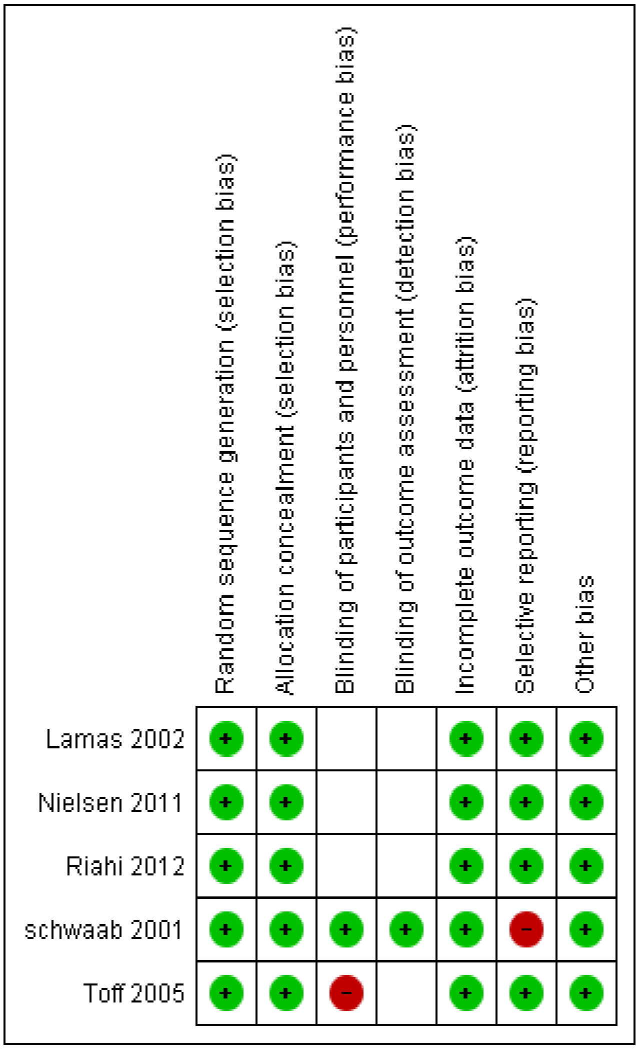

Supplementary Table 4: Quality Assessment Using Newcastle Ottawa Scale

	Selection				Comparability	Outcome			
**Study**	**Representativeness of exposed cohort (Maximum: *)**	**Selection of the non-exposed cohort (Maximum:*)**	**Ascertainment of exposure (Maximum:*)**	**Outcome at the start (Maximum:*)**	**Comparability of cohorts on the basis of the design or analysis (Maximum:**)**	**Assessment of outcome (Maximum: *)**	**Adequate follow-up length (Maximum: *)**	**Adequate follow-up (Maximum: *)**	**Total (out of 9)**
**Huang 2003**	*	*	*	*	*	*	_	*	7
**Wiegand 2003**	*	*	*	*	*	*	*	*	8
**Masumoto 2004**	*	*	*	*	**	*	*	*	9
**Lin 2007**	*	*	*	*	_	*	*	*	7
**Kim 2010**	*	*	*	*	**	*	*	*	9
**Marchandise 2011**	*	*	*	*	*	*	*	*	8

Supplementary Table 5: Eggers Test of Included RCT

Outcomes	P values
Stroke	0.81
All-cause mortality	0.49
Heart Failure	0.86
Atrial Fibrillation	0.009

Supplementary Table 6: Eggers Test of Included Observational Studies

Outcomes	P-values
Atrial Fibrillation	0.06
Congestive Heart Failure	0.23
All-cause Mortality	0.94
Overall Complication	0.96
Pneumothorax	0.18
Atrial Lead Dislodgement	0.22

Figure 2: Funnel Plots of Included Clinical Trials

2A

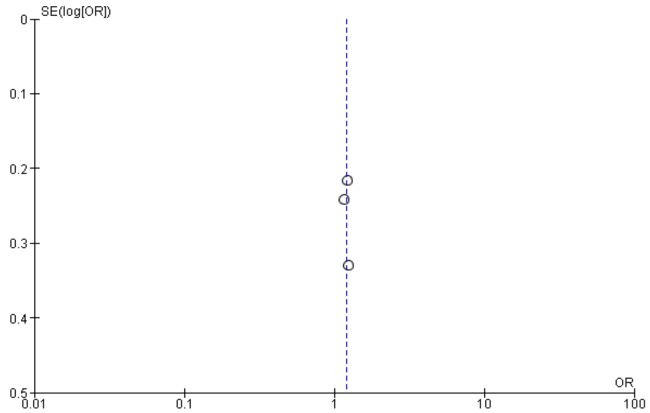

2B

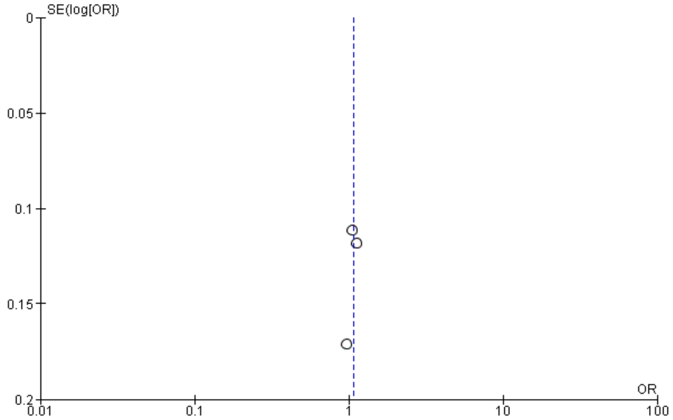

2C

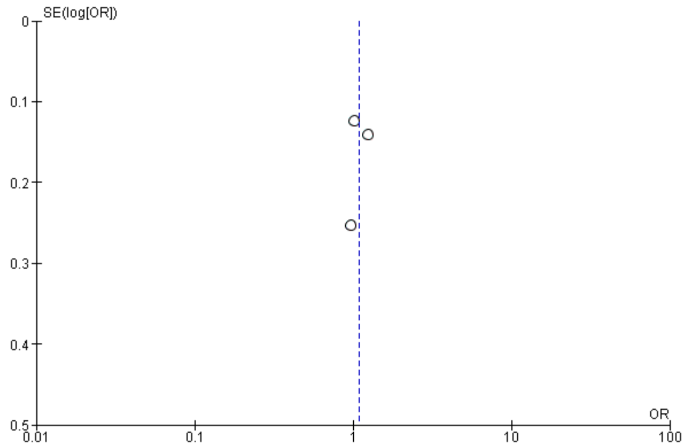

2D

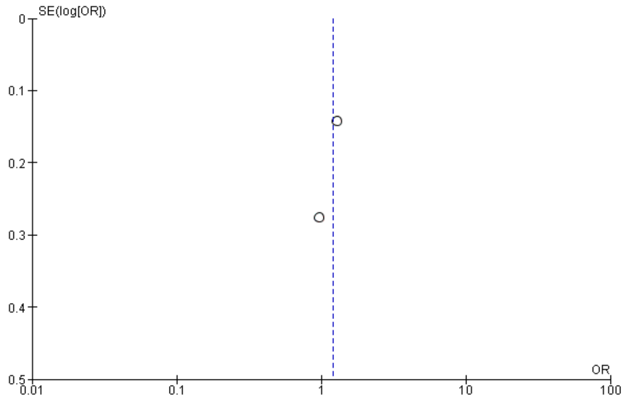

2E

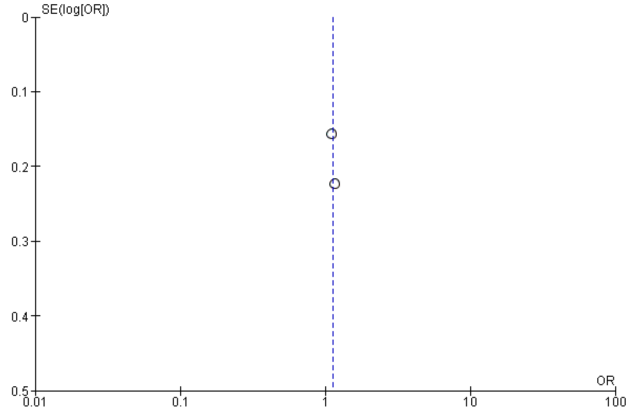

2F

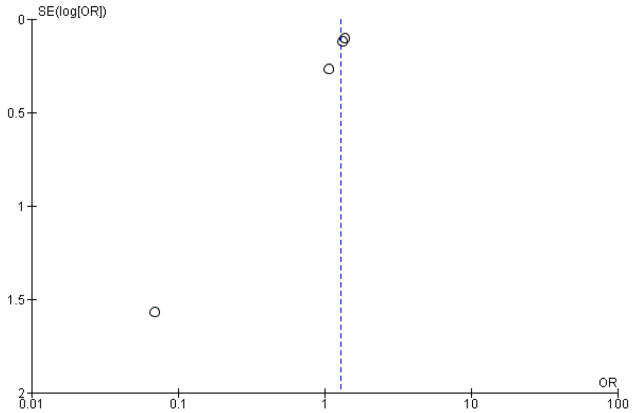

Figure 3: Funnel Plots of Included Observational Studies

3A

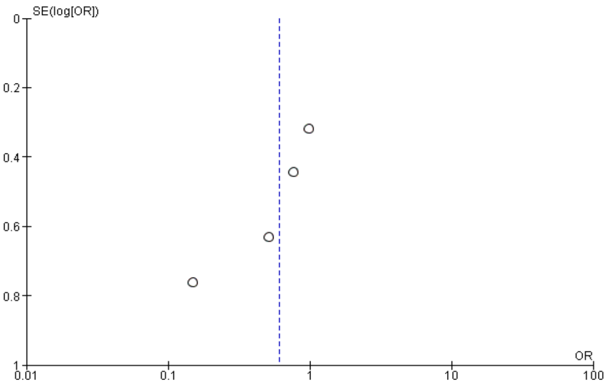

3B

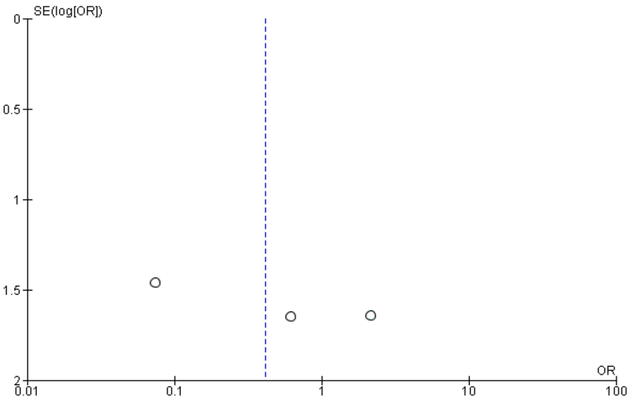

3C

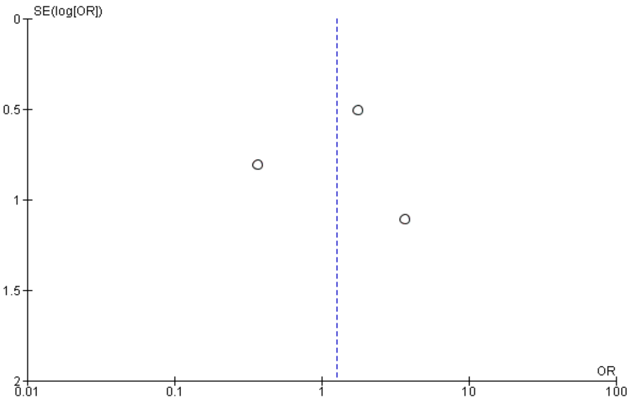

3D

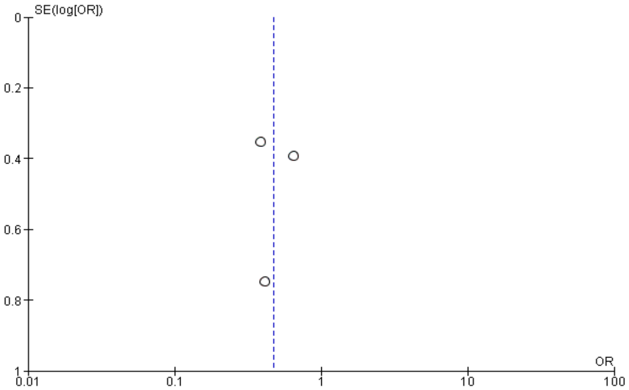

3E

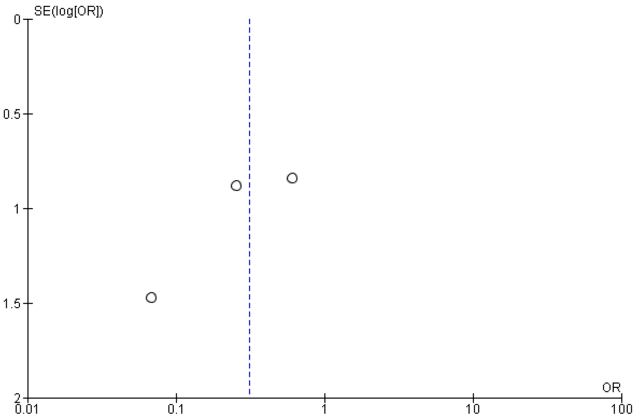

3F

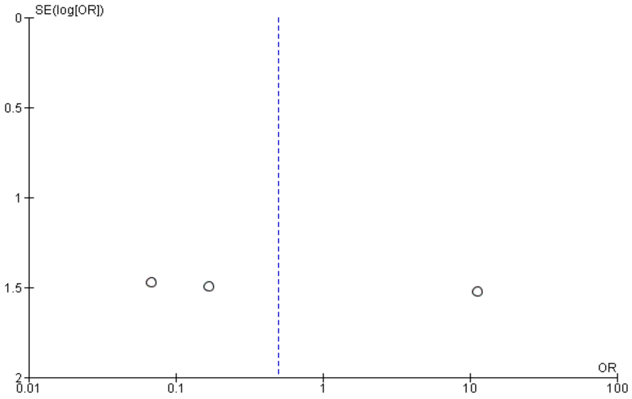

Supplement: Multimedia component 1 [file mmc1.pdf]
